# Supplementary material for: Stacking Model‐Based Classifiers for Dealing With Multiple Sets of Noisy Labels
Source: Biom J. 2025 Mar 12;67(2):e70042. doi: 10.1002/bimj.70042 (PMC11898607; doi:10.1002/bimj.70042)
Supplement: Supplementary file 1 — Supporting Information [file BIMJ-67-e70042-s001.zip › density-based-ensemble-model-main/competitors_real_data/netlab-master/netlab-master/help/evidence.htm]

Netlab Reference Manual evidence


# evidence

## Purpose

Re-estimate hyperparameters using evidence approximation.

## Synopsis

```
[net] = evidence(net, x, t)
[net, gamma, logev] = evidence(net, x, t, num)
```

## Description

`[net] = evidence(net, x, t)` re-estimates the
hyperparameters `alpha` and `beta` by applying Bayesian
re-estimation formulae for `num` iterations. The hyperparameter
`alpha` can be a simple scalar associated with an isotropic prior
on the weights, or can be a vector in which each component is
associated with a group of weights as defined by the `index`
matrix in the `net` data structure. These more complex priors can
be set up for an MLP using `mlpprior`. Initial values for the iterative
re-estimation are taken from the network data structure `net`
passed as an input argument, while the return argument `net`
contains the re-estimated values.

`[net, gamma, logev] = evidence(net, x, t, num)` allows the re-estimation
formula to be applied for `num` cycles in which the re-estimated
values for the hyperparameters from each cycle are used to re-evaluate
the Hessian matrix for the next cycle. The return value `gamma` is
the number of well-determined parameters and `logev` is the log
of the evidence.

## See Also

`mlpprior`, `netgrad`, `nethess`, `demev1`, `demard`

---

**Pages:**
Index


---

Copyright (c) Ian T Nabney (1996-9)
